# The Heme Oxygenase/Biliverdin Reductase System as Effector of the Neuroprotective Outcomes of Herb-Based Nutritional Supplements

**DOI:** 10.3389/fphar.2019.01298

**Published:** 2019-11-11

**Authors:** Emanuela Mhillaj, Vincenzo Cuomo, Luigia Trabace, Cesare Mancuso

**Affiliations:** ^1^Institute of Pharmacology, Università Cattolica del Sacro Cuore, Roma, Italy; ^2^Department of Physiology and Pharmacology “V. Erspamer,” Sapienza University of Rome, Rome, Italy; ^3^Department of Clinical and Experimental Medicine, University of Foggia, Foggia, Italy; ^4^Fondazione Policlinico Universitario A. Gemelli IRCCS, Roma, Italy

**Keywords:** ferulic acid, *Ginkgo biloba*, heme oxygenase, neuroprotection, resveratrol

## Abstract

Over the last few years, several preclinical studies have shown that some herbal products, such as ferulic acid, *Ginkgo biloba*, and resveratrol, exert neuroprotective effects through the modulation of the heme oxygenase/biliverdin reductase system. Unfortunately, sufficient data supporting the shift of knowledge from preclinical studies to humans, particularly in neurodegenerative diseases, are not yet available in the literature. The purpose of this review is to summarize the studies and the main results achieved on the potential therapeutic role of the interaction between the heme oxygenase/biliverdin reductase system with ferulic acid, *G. biloba*, and resveratrol. Some critical issues have also been reported, mainly concerning the safety profile and the toxicological *sequelae* associated to the supplementation with the herbs mentioned above, based on both current literature and specific reports issued by the competent Regulatory Authorities.

## Background

At the end of Sixties, three skilled scientists from the University of California San Francisco Medical Center first described and characterized the enzymatic activity of heme oxygenase (HO), a microsomal enzyme catalyzing the oxidative cleavage of hemoproteins’ prosthetic group in equimolar amounts of ferrous iron, carbon monoxide (CO) and biliverdin (BV) (Tenhunen et al., 1968; Tenhunen et al., 1969). Following this initial description of the enzymatic activity, there were many other findings, including the inducible and constitutive nature of HO (HO-1 and HO-2, respectively), as well as the full characterization of biliverdin reductase (BVR), a cytosolic enzyme that works in combination with HO and reduces BV into bilirubin (BR). A significant contribution to these discoveries was provided by Mahin Maines and her research group [see (Maines, 1988; Maines, 1997; Maines, 2005) and references therein] who, recently, have deepened the field by describing pleiotropic effects of BVR in terms of modulation of numerous cytoprotective signaling pathways [see (Maines and Gibbs, 2005; Kapitulnik and Maines, 2009; Gibbs et al., 2015) and references therein].

Following these observations, the HO/BVR system has been studied worldwide by scientists who have gradually discovered its enormous potential. Just to give an idea of the attention aroused, suffice it to say that since 1969, more than 15300 papers have been published containing the keyword “heme oxygenase” (source: PubMed, accessed on August 8, 2019), while, concerning “biliverdin reductase,” the first studies appeared in 1965 reaching, to date, more than 450 papers (source: PubMed, accessed on August 8, 2019). The numbers mentioned so far have been achieved thanks to the many studies that have described the involvement of the HO/BVR system in the pathogenesis of Alzheimer’s disease (AD), Parkinson’s disease (PD), atherosclerosis and other cardiovascular disorders, kidney diseases, diabetes, etc. The molecular mechanisms through which the HO-1/BVR system exerts neuroprotective effects mainly depends on the down-stream effectors CO and BR. In the nervous system, CO has been shown to modulate synaptic plasticity, neuropeptide secretion, and neurogenesis (Verma et al., 1993; Mancuso et al., 1998; Mancuso et al., 2010; Choi, 2018), whereas BR interacts with reactive oxygen species (ROS), reactive nitrogen species (RNS) and nitric oxide (NO) to prevent neurotoxicity due to free radical injury (Mancuso et al., 2006a; Mancuso et al., 2008; Barone et al., 2009b; Mancuso et al., 2012a). While the antioxidant and cytoprotective effects of the HO/BVR system and its by-products have been extensively described in literature, scarce attention has been dedicated to a critical examination of their potentially toxic effects. Our research group has contributed to this field by describing the cytotoxic effects of CO either through the inhibition of the stress axis under pro-inflammatory conditions or production of pro-inflammatory prostaglandins (PG) (e.g., PGE2) in rat hypothalamus [see (Mancuso et al., 1997; Mancuso et al., 2006c; Mancuso et al., 2010) and references therein]. Moreover, we have also explored the cytoprotective *vs* cytotoxic effects of the interaction between BR and NO [see (Mancuso et al., 2006b; Mancuso, 2017) and references therein; (Barone et al., 2009b)]. Hyman Schipper’s group, instead, decisively contributed to the discovery of the neurotoxic role of HO-derived iron in mitochondrial dysfunctions and in the genesis of oxidative stress-induced damage in neurons and glial cells [see (Schipper, 2004; Schipper et al., 2009; Schipper et al., 2019) and references therein]. Finally, Shigeki Shibahara and Kazuhiko Igarashi, with collaborators, discovered the importance of HO-1 gene repression, through the transcription factor Bach1, to reduce cellular toxic effects due to iron and CO accumulation as in the case of strong and long-lasting pro-oxidant conditions (Shibahara, 2003; Shibahara et al., 2003; Igarashi and Sun, 2006). These findings contributed to increase the awareness of the risks associated with the uncontrolled activation of the HO/BVR system.

A particularly detailed aspect, starting from the early 2000s, was the interaction between HO-1 and products of herbal origin, and caffeic acid derivatives were the first to be studied (Scapagnini et al., 2002); only later, many studies have appeared in the literature concerning the ability of several other herbal products to induce HO-1 (Mancuso et al., 2012b; Mancuso and Santangelo, 2014; Fetoni et al., 2015; Mancuso and Santangelo, 2017). However, as detailed below, the up-regulation of HO-1 by herb-derived nutritional supplements is claimed as a beneficial mechanism through which they exert neuroprotective outcomes. In this regard, however, it is worth underlining how not always the fine involvement of HO-1 in the claimed neuroprotective effects of herbal products has been studied, but frequently HO-1 induction has been considered rather as a mere biomarker of activation of the cell stress response. In our opinion, the aspects that are still weak and are worth investigating, with regard to the effects of herbal products through HO-1 up-regulation, are the following: (i) the correlation between the dose/concentration of herbal product capable of inducing HO-1 *in vitro* and its effective concentration in the target organ within *in vivo* studies; (ii) the extent and duration of HO-1 induction, considering that, as previously mentioned, the accumulation of products, such as iron and CO can become toxic to cells, (iii) loss of specificity of the obtained results, as nearly all the studied herbal products induced HO-1 and (iv) the reduced number of studies that evaluated the BVR modulation, through herbal products.

The purpose of this review is to provide a critical overview of the potential therapeutic role of ferulic acid, *Ginkgo biloba*, and resveratrol *via* the modulation of the HO/BVR system; the reason why the attention has been focused on these herbal products depends on the consistent number of articles published on this topic through the years strong enough to substantiate a potential interest towards clinics. The safety profile of ferulic acid, *G. biloba*, and resveratrol has been also evaluated with the purpose to provide a complete overview of the risk/benefit balance of a chronic supplementation with these agents.

## Ferulic Acid

**Figure 1:**
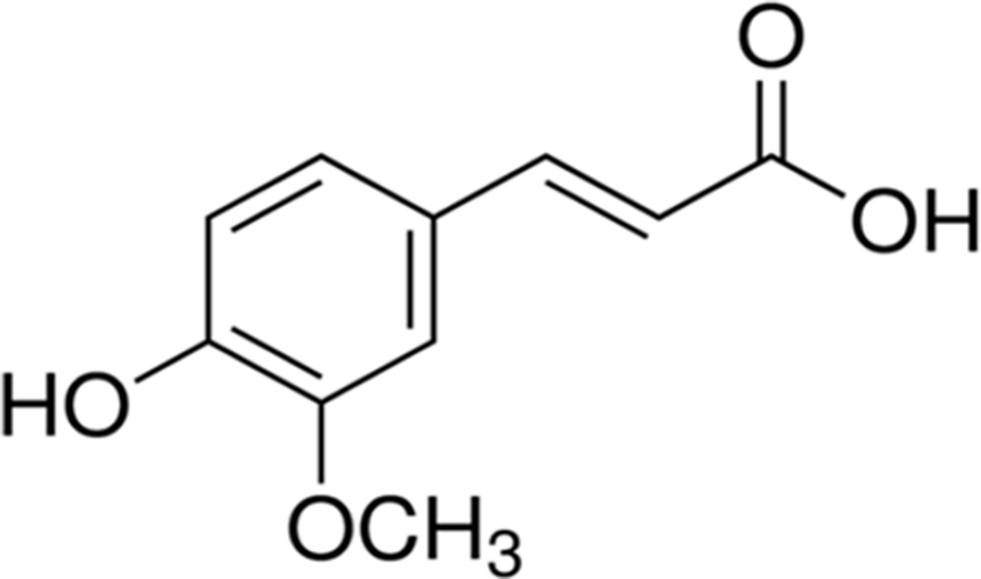
The chemical structure of ferulic acid.

Ferulic acid {[(E)-3-(4-hydroxy-3-methoxy-phenyl)prop-2-enoic acid)], FA, **Figure 1**} belongs to the family of phenolic acids and is highly abundant in fruits and vegetables. Furthermore, FA is also a component of Chinese medicinal herbs, such as *Angelica sinensis*, *Cimicifuga racemosa*, and *Ligusticum chuangxiong*. The main pharmacokinetic parameters of FA are shown in **Table 1**.

**Table 1:** Main pharmacokinetic parameters for ferulic acid, *Ginkgo biloba*, and resveratrol in humans.

	Bioavail. (%)	*T*_max_ (h)	*T*_1/2_ (h)	Excretion	References
Ferulic acid *per os*	20%	0.4–3	0.7–5	Urine (glucuronide, sulfoglucuronide, and glycine metabolites)	(Mancuso and Santangelo, 2014)
*G. biloba* ginkgolides bilobalide *per os*	> 80%	0.5–3	4–10	Urine (40–70% unchanged)	(Kleijnen and Knipschild, 1992; Ude et al., 2013)
	∼ 70%	0.5–3	3–5	Urine (30% unchanged)	
Resveratrol *per os*		1–1.5 6*	9–11	Urine (monoglucuronide and sulfate metabolites)	(Wang and Sang, 2018)

*Second peak probably due to the enteric recirculation of conjugate metabolites.

Bioavail., biovailability; T_max_, time to reach peak plasma concentration; T_1/2_, half-life.

Over the past years, several studies have shown that FA acts as a strong antioxidant not only by a direct free radical-scavenging mechanism, but largely by enhancing the cell stress response through the up-regulation of the HO/BVR system (Barone et al., 2009a; Mancuso and Santangelo, 2014). On the other hand, literature data have highlighted the contribution of FA-induced HO-1 up-regulation to numerous biological effects in several preclinical models (see **Table 2**).

**Table 2:** Contribution of HO-1 up-regulation to the biological effects of ferulic acid (FA) in preclinical *in vitro* and *in vivo* models.

Preclinical model	Ferulic acid* (concentration or dose)	Effect(s)	Reference(s)
Radiation-induced damage in mice	50 mg/kg *per os* for 5 days	Prevention of radiation-induced oxidative damage in the duodenum	(Das et al., 2017)
Cisplatin-induced nephrotoxicity in rats	50 mg/kg *per os* for 5 days	Prevention of drug-induced injury and improvement of renal function	(Bami et al., 2017)
Pre-adipocytes	100 µM	Reduction of adipocyte tissue mass	(Koh et al., 2017)
Lymphocytes	0.001–0.1 µM	Inhibition of oxidative damage.	(Ma et al., 2011)
Endothelial cells	0.2–5 µM	Prevention of radiation-induced oxidative damage	(Ma et al., 2010)
Melanocytes	1–50 µM^†^	Prevention of UVB-induced skin oxidative damage	(Di Domenico et al., 2009)
Rat heart	100 mg/kg *per os* for 14 days	Increase of the antioxidant defense in cardiac tissue	(Yeh et al., 2009)
Dermal fibroblasts	25 µM^†^	Prevention of hydrogen peroxide-induced oxidative damage.	(Calabrese et al., 2008)

*Only studies carried out with purified FA or congeners have been included in this table.

^†^Ferulic acid ethyl ester.

UVB, ultraviolet B.

Ferulic acid and its ethyl ester [5–50 µM or 150 mg/kg intraperitoneal (i.p.)] were reported to over-express HO-1 in rat neurons and gerbil synaptosomes resulting in a significant neuroprotective effect on ROS- and glucose oxidase- related oxidative damage (Kanski et al., 2002; Scapagnini et al., 2004; Joshi et al., 2006). In the neuronal cell line SH-SY5Y, FA (1–10 µM) exerted marked neuroprotective effects against trimethyltin-induced damage by increasing the expression of HO-1; the translocation of the transcriptional inducer nuclear factor erythroid 2-related factor 2 (Nrf2) from cytosol to the nucleus has been described as the molecular mechanism underlining the FA-induced HO-1 up-regulation (Catino et al., 2016). Moreover, CO and BR have been identified as the by-products of HO and BVR activities responsible for the antioxidant and neuroprotective effects of FA (Catino et al., 2016). Through the specific up-regulation of the Nrf2/HO-1 system, FA (3–30 µM) counteracted lead-induced inhibition of neurite outgrow in PC12 cells (Yu et al., 2016). As shown by Ma et al. (Ma et al., 2011), the extracellular signal-regulated kinase (ERK) has a role in mediating the FA-activation of Nrf2/HO-1 since its blockade counteracts the nuclear translocation and transcriptional activity of Nrf2 on the HO-1 gene. Quite recently, our research group has demonstrated how FA exerts neuroprotective effects not only under pro-oxidant conditions, but also during psychosocial stress. As shown by Mhillaj et al. (Mhillaj et al., 2018), FA (150 mg/kg i.p.) enhances long-term memory in rats exposed to novelty-induced emotional arousal through the up-regulation of HO-1 in the hippocampus and frontal cortex; it is worth mentioning the finding that FA also over-expressed HO-2 in the frontal cortex. CO is responsible for this nootropic effect of FA, whereas BV does not have any significant effect (Mhillaj et al., 2018).

Ferulic acid (150 mg/kg i.p. for 4 days) up-regulated HO-1 in the organ of Corti of guinea pigs exposed to acoustic trauma; FA-induced improvement of the auditory function was counteracted by the HO inhibitor zinc-protoporphyrin-IX and paralleled the time-course of FA-induced HO-1 overexpression, thus supporting the hypothesis that the neuroprotective effect of this phenolic acid was due to the induction of cytoprotective HO-1 (Fetoni et al., 2010). Interestingly, FA (21.61 mg/kg for 3 weeks intragastric) increased the HO-1 expression and counteracted visible light-induced retinal degeneration in pigmented rabbits (Wang et al., 2016b).

Ferulic acid has also been complexed with tacrine, one of the earliest drugs developed for AD therapy and later discarded for severe hepatotoxicity. Tacrine-FA (2–100 µM) has been shown to prevent β-amyloid (Aβ) aggregation, ROS formation, and apoptosis in PC12 cells; in addition, tacrine-FA (2–20 mg/kg intragastric) improved cognitive skills in a mouse model of AD (Pi et al., 2012). In a subsequent paper, Huang et al. (Huang et al., 2012) showed how tacrine-FA inhibits oxidative stress-induced damage by over-expressing HO-1, through Nrf2 translocation, in HT22 cells.

Ferulic acid has an enviable safety profile, since no significant toxicities have been reported in humans; furthermore, FA’s modulation of drug metabolizing enzymes is negligible (see https://toxnet.nlm.nih.gov. Accessed on June 27, 2019). However, female rats treated with the highest tolerated dose of tacrine-FA have shown both glycogen depletion and HO-1 induction and cytochrome-P450 (CYP) CYP1A1, 2B1, and 3A2 up-regulation in the liver, mainly due to the tacrine moiety (Lupp et al., 2010). A careful risk/benefit analysis of the pharmacological and safety profiles of tacrine-FA is required.

## *Ginkgo Biloba*

**Figure 2:**
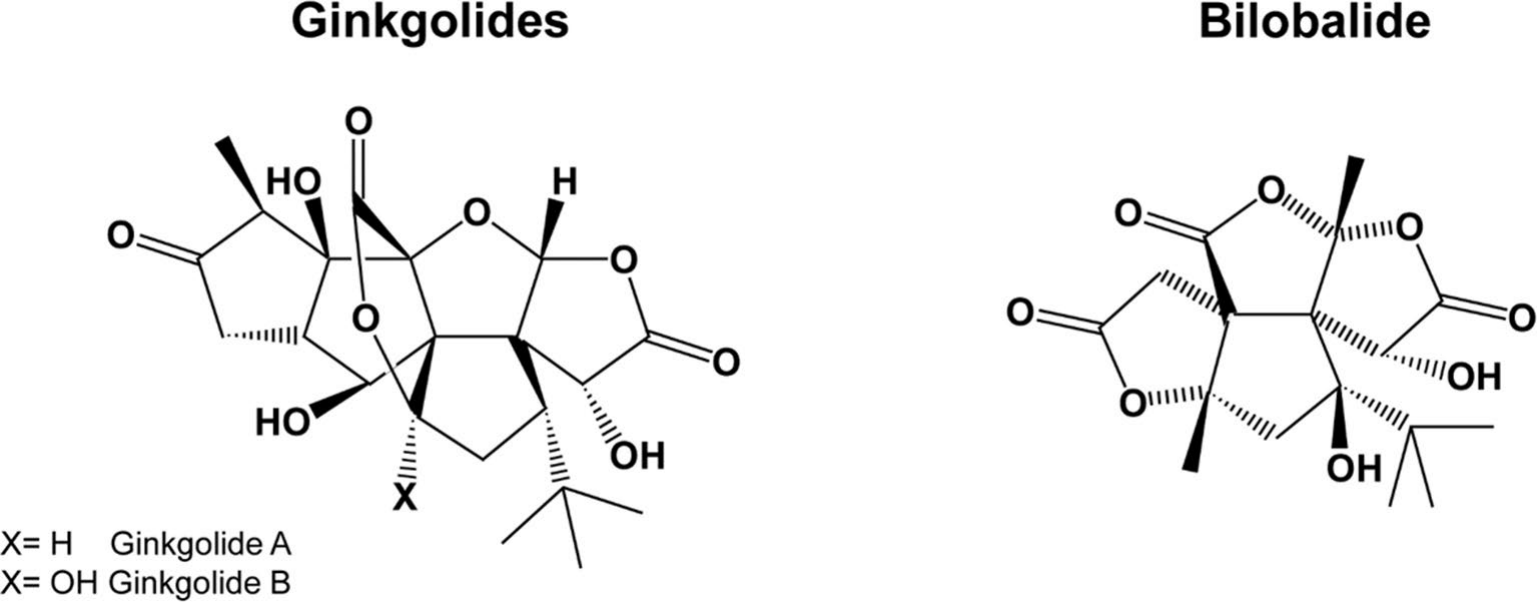
The chemical structure of ginkgolides and bilobalide.

*Gingko biloba* is a plant growing in the mountainous valleys of Eastern China. *G. biloba* extracts contain several active compounds, including flavonoids, terpenes, organic acids, and polyphenols, the most important being flavonol-glycosides (primarily quercetin, kaempferol, and isorhamnetin) and terpene-lactones; the latter are further divided into diterpenes (ginkgolides) and sesquiterpenes (bilobalide) (**Figure 2**). For a summary of the pharmacokinetic parameters and biological effects of *G. biloba*, see **Tables 1** and **3**, respectively.

**Table 3:** Contribution of HO-1 up-regulation to the biological effects of *Ginkgo biloba* in preclinical *in vitro* and *in vivo* models.

Preclinical model	*G. biloba* (concentration or dose)	Effect(s)	Reference(s)
Myoblasts	25–100 µg/mL	Cytoprotection from alcohol-induced oxidative damage	(Wang et al., 2015)
Endothelial cells	50–200 µg/mL	Endothelial protection from high-glucose- or TNF-α–induced vascular oxidative damage; cytoprotection against cigarette smoke-induced apoptosis in lungs	(Hsu et al., 2009; Chen et al., 2011; Tsai et al., 2013)
Macrophages	1–100 µg/mL	Inhibition of inflammatory damage in LPS-treated cells; regulation of cholesterol homeostasis and reduction in atherosclerosis lesion size	(Tsai et al., 2010; Ryu et al., 2012)
Ethanol-induced liver damage in rats	48 or 96 mg/kg intragastric for 90 days	Reduction of oxidative damage and improvement of ethanol-induced microvesicular steatosis and parenchimatous degeneration in hepatocytes	(Yao et al., 2007)

LPS, lipopolysaccharide; TNF, tumor necrosis factor.

Through the induction of HO-1, ginkgolides A, B, and C [1, 3, and 10 mg/kg by intravenous route (i.v.)] decreased neurological deficits and brain infarct volume in rats exposed to ischemia/reperfusion damage (Zhang et al., 2018). In a similar experimental setting the *G. biloba* extract EGb 761 [containing flavonol-glycosides (24%), ginkgolides A, B, and C (2.8–3.4%), and bilobalide (2.6–3.2%)] at the dosage of 100 mg/kg *per os* for 7 days before or 4-24 h after damage reduced cortical infarct volume and stimulated the proliferation of neuronal stem/progenitor cells *via* HO-1 overexpression in mice with permanent middle cerebral artery occlusion (Shah et al., 2011; Nada and Shah, 2012; Nada et al., 2014). The same research group confirmed the neuroprotective effects of *G. biloba* in the hippocampus of mice pre-treated with EGb 761 (100 mg/kg *per os* for 7 days) and then susceptible to bilateral common carotid artery occlusion (Tulsulkar and Shah, 2013). The contribution of HO-1 to the neuroprotective effects of EGb 761 (100 mg/kg *per os*) was also demonstrated in HO-1 knockout male mice with middle cerebral artery occlusion (Saleem et al., 2008). Ginkgolide B (1–50 µM or 10 mg/kg i.p.) prevented cisplatin-induced damage in HEI-OC1 auditory cells and rats through HO-1 induction (Ma et al., 2015). Interestingly, in order to improve brain penetration, *G. biloba* has been complexed with phosphatidylcholine (Carini et al., 2001); this novel formulation exhibited neuroprotective effects by increasing catalase, superoxide dismutase, glutathione peroxidase, and glutathione reductase activities in rat brain (Naik et al., 2006). Regrettably, there are no studies addressing the potential role of this novel formulation on HO-1. Worth mentioning is the nootropic effects of a novel formulation of *G. biloba* (120 mg/day *per os*) complexed with phosphatidylserine administered to healthy volunteers for 7 days (Kennedy et al., 2007).

As far as neurodegenerative disorders, no evidence has been found in literature dealing with the involvement of HO-1 and/or BVR in *G. biloba*-related neuroprotection. Indeed, the neuroprotective effects of EGb 761 have been extensively reported in both AD (Augustin et al., 2009; Liu et al., 2015; Wan et al., 2016) or PD rodent models (Kim et al., 2004; Rojas et al., 2012; El-Ghazaly et al., 2015) and in humans. Over the last 2–3 years, some retrospective analyses, meta-analyses, and systematic reviews on the neurotherapeutic effects of *G. biloba* extracts in subjects with dementia, including AD, have been published and the conclusions are the following: (i) over 12 months treatment, EGb 761 and donepezil showed similar effects in cognitive decline in patients aged 80 years or older affected by AD (Rapp et al., 2018); (ii) EGb 761 (240 mg/day *per os* for 22–24 weeks) had a better performance than placebo in 1,598 patients with dementia (probable AD with or without cerebrovascular disease and probable vascular dementia) (Savaskan et al., 2018); (iii) doses lower than 200 mg/day did not have any remarkable clinical effects in demented people (Yuan et al., 2017) and (iv) *G. biloba* extract (240 mg/day *per os*) also showed neuroprotective effects in subjects with mild cognitive impairment (Zhang et al., 2016; Kandiah et al., 2019). Unfortunately, there are no clinical studies on the neuroprotective effects of *G. biloba* in PD patients, therefore no conclusions can be drawn.

Although the safety profile of *G. biloba* extracts is acceptable, few mild adverse effects, including mild gastrointestinal complaints, headaches, and allergic reactions have been reported (Kleijnen and Knipschild, 1992). However, important interactions between *G. biloba* and common drugs should be underlined. *G. biloba* is an inducer of CYP2C19 and, through this mechanism, it has been shown to reduce omeprazole plasma levels in individuals sharing the poor metabolizer phenotype (Yin et al., 2004) and to increase metabolism and reduce plasma concentrations of the antiepileptic drugs, valproic acid, and phenytoin, thus increasing the risk of fatal seizures (Kupiec and Raj, 2005). A greater risk of bleeding has been reported in people taking *G. biloba* and aspirin or warfarin (Agbabiaka et al., 2017). *G. biloba* has been discouraged in people taking selective serotonin-reuptake inhibitors for the increased risk of developing serotonin syndrome (Kreijkamp-Kaspers et al., 2015). Lastly, ginkgo flavonol-glycosides caused a coma in an 80-year-old AD patient taking trazodone, probably by stimulating the CYP3A4 activity which increases the transformation of trazodone into the active metabolite 1-(m-chlorophenyl) piperazine and up-regulates GABAergic activity in the brain (Galluzzi et al., 2000).

## Resveratrol

**Figure 3:**
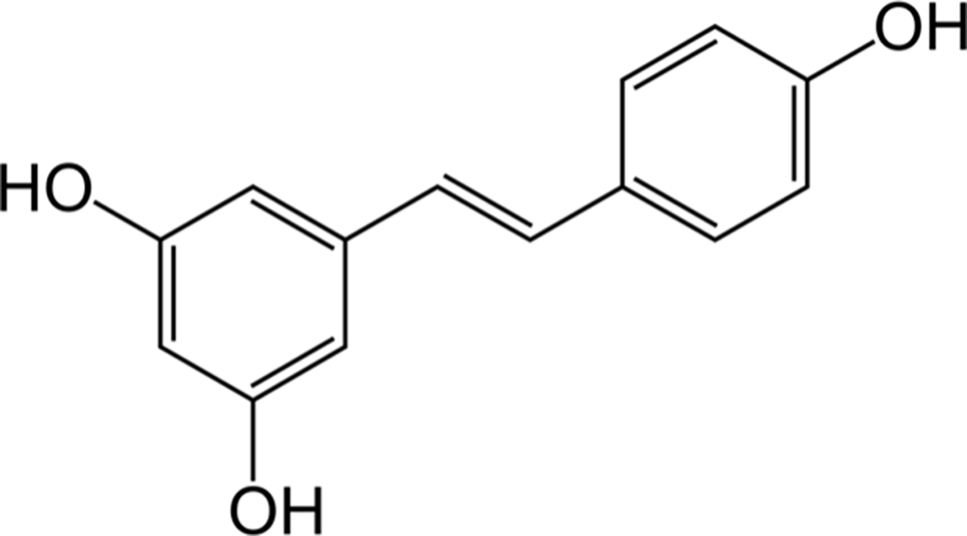
The chemical structure of resveratrol.

Resveratrol (3,5,4’-trihydroxy-*trans*-stilbene, (**Figure 3**) is a phytoalexin found in grapes, cranberries, peanuts, and some beverages (Mancuso et al., 2007). However, wine is considered the main source of resveratrol since the solubility of the latter in ethanol is about 1,667-times higher than that in water (Weiskirchen and Weiskirchen, 2016). Also based on the interest aroused by the so-called “French paradox” over the last few years, the interaction resveratrol-HO-1 has been extensively studied for its beneficial effects in several preclinical models of disease (**Table 4**).

**Table 4:** Contribution of HO-1 up-regulation to the biological effects of resveratrol in preclinical *in vitro* and *in vivo* models.

Preclinical model	Resveratrol (concentration or dose)	Effect(s)	Reference(s)
Kidney injury in rats	30 mg/kg i.p.	Amelioration of sepsis-induced kidney injury	(Wang et al., 2018a)
Renal cells	20 µM	Cytoprotection from nicotine-induced oxidative damage.	(Arany et al., 2017)
Lung injury in rodents	30 mg/kg i.p. 1–3 mg/kg *per os* for 3 days	Improvement of sepsis- or paraquat-induced lung injury in rats Enhancement of cell stress response and attenuation of cigarette smoke-induced damage in mice	(Li et al., 2016; Wang et al., 2018b) (Liu et al., 2014)
Renal carcinoma in rats	30 mg/kg *per os* for 24 weeks	Inhibition of proliferation and improvement of renal function; increase in the antioxidant system	(Kabel et al., 2018)
Membranous nephropathy in mice	30 mg/kg s.c. every other day for 6 weeks	Reduction of apoptosis and complement-induced damage; amelioration of renal function	(Wu et al., 2015)
Endothelial cells	0.01–10 µM	Reduction of oxidative stress-induced damage and inhibition of senescence in progenitor cells	(Shen et al., 2016b)
Smooth muscle cells	1–10 µM	Inhibition of oxidative damage and inflammation; vascular protection	(Juan et al., 2005)
Obstructive jaundice in rats	10–20 mg/kg *per os*	Restoration of intestinal permeability and improvement of gut barrier function	(Wang et al., 2016a)
Gastric inflammation in mice	100 mg/kg *per os* for 6 weeks	Reduction of oxidative damage and inflammation in *Helicobacter pylori*–infected gastric mucosa	(Zhang et al., 2015)
Myocardial damage in rats	100 µM i.v.	Reduction of oxidative damage and improvement of cardiac function following ischemia/reperfusion injury	(Cheng et al., 2015)
Hepatoma cells	1 µM	Stimulation of mitochondrial biogenesis and reduction of inflammatory damage	(Kim et al., 2014)
Macrophages	1–10 µM	Inhibition of inflammatory damage	(Son et al., 2014)

i.p., intraperitoneal route of administration; i.v., intravenous route of administration; s.c., subcutaneous route of administration.

With regard to the neuroprotective effects, earlier studies showed that resveratrol (5–100 µM) up-regulates HO-1 in primary cultures of mouse neurons (Zhuang et al., 2003). Resveratrol (15 µM), was also found to increase HO-1 expression in neuron-like PC12 cells through both phosphoinositide 3-kinase and MEK1/2 activities (Chen et al., 2005). Quite recently, resveratrol (10 µM) has exhibited a marked protective effect on primary rat oligodendrocyte progenitor cells exposed to lipopolysaccharide through the modulation of Nrf2/HO-1 pathway and the enhancement of cell stress response (Rosa et al., 2018). Neuroprotective effects, due to HO-1 up-regulation, have also been shown in C6 astroglial cells and in hippocampal primary rat astrocytes treated with 100 µM resveratrol and then exposed to neurotoxicants, such as buthionine sulfoximine, azide, or ammonia, for 3–24 h (Bobermin et al., 2015; Bellaver et al., 2016; Arus et al., 2017).

Resveratrol (20–40 mg/kg i.p. for 7 days) significantly enhanced HO-1 expression and prevented both cerebral edema and infarction in seven-day-old rat pups exposed to hypoxic/ischemic injury secondary to unilateral carotid artery ligation (Gao et al., 2018). Similar results have been obtained in adult male rats treated with resveratrol (15–30 mg/kg i.p. for 7 days) and undergoing middle cerebral artery occlusion; in these animals, HO-1 induction was paralleled by down-regulation of caspase-3 and improvement of neuronal viability (Ren et al., 2011). Resveratrol (1–100 µM) increased HO-1 expression in both rat neural stem cells and primary rat cortical neurons exposed to glucose deprivation/reoxygenation injury (an *in vitro* experimental model mimicking cerebral artery occlusion and reperfusion injury) along with a strong enhancement of cell stress response and a marked reduction of apoptotic cell death (Shen et al., 2016a; Yang et al., 2018). As far as AD is concerned, resveratrol (10–40 µM) has been shown to counteract Aβ-induced oxidative stress in PC12 cells through the overexpression of Nrf2/HO-1 system (Hui et al., 2018). In Aβ-treated rats, resveratrol [100 µM/5 µL by intracerebroventricular route (i.c.v.)] up-regulated hippocampal HO-1, reduced neuronal death in the same area, and improved spatial memory (Huang et al., 2011). In SH-SY5Y cells exposed to rotenone, resveratrol (10–20 µM) induced HO-1 expression and prevented dopaminergic cell death by autophagy (Lin et al., 2014).

Studies in humans have found that resveratrol (25 mg *per os*) has a low bioavailability and reaches plasma concentrations of about 40 nM within 2 h from administration (Goldberg et al., 2003; Walle et al., 2004); plasma concentrations up to 2.4 µM are reached if the dose of resveratrol increases up to 1 g (Almeida et al., 2009). For a summary of resveratrol pharmacokinetics see **Table 1**. In order to improve bioavailability, resveratrol has been complexed with solid lipid nanoparticles (SLN) or gold-conjugated nanoparticles (Park et al., 2016; Yadav et al., 2018). Among these formulations, SLN increased by about 4-times the brain levels of resveratrol, which was able to up-regulate HO-1 and improve cognitive decline in rats with permanent bilateral common carotid artery occlusion (Yadav et al., 2018). Unfortunately, there are no studies in humans on the effective concentrations of resveratrol in the brain and a brain concentration greater than 2.4 µM is unlikely, by considering the plasma concentrations reached in the studies mentioned above and the presence of the blood-brain barrier. This suggests that most of the preclinical studies on the neuroprotective effects of resveratrol cannot be applied to humans. As far as the role of wine as the source of resveratrol is concerned, considering both the annual consumption of red and white wines in France (31.7 vs 11.7 L, respectively) and the amount of resveratrol contained in red and white wines (2 and 0.5 mg/L, respectively), it is possible to calculate an amount of resveratrol “drunk” of about 0.2 mg/day, 5000-times less than the highest daily dosage (1 g/day) claimed to give rise to pharmacological effects (Weiskirchen and Weiskirchen, 2016).

It is worth mentioning the document released by the EFSA regarding the request by the European Commission on the safety of trans-resveratrol as a novel food (Efsa, 2016). In this document, the EFSA established that the 38 clinical studies provided in support of the claim do not lead to any conclusion about the efficacy of resveratrol, at doses up to 5 g/day for either acute (4 days) or chronic administration (4–12 weeks), in the treatment of metabolic diseases or cancer. The EFSA pointed out the heterogeneity of the resveratrol doses, the low number of individuals recruited, the uncontrolled experimental design, and the exploratory character of several studies.

In some clinical studies, patients supplemented with resveratrol reported mild adverse effects, such as diarrhea and hot flushes. As far as the interaction with drugs-metabolizing enzymes is concerned, resveratrol has been shown to inhibit CYP3A4, CYP2D6, and CYP2C9 and to induce CYP1A2 *in vitro*. No significant changes in plasma levels of common drugs have been reported so far (Efsa, 2016).

## Conclusions

In illustrating the interactions between the HO/BVR system with FA, *G. biloba*, and resveratrol, we have attempted to address some critical points presented in the Introduction, discussing each issue to the best of our knowledge.

An initial consideration concerns the janus face of the HO-1/BVR by-products. As mentioned in the Introduction, both CO and BR have important pleiotropic and neuroprotective effects in the nervous system, but they may become toxic in the case of a disproportionate production or under conditions of redox imbalance (Mancuso et al., 1997; Mancuso et al., 2010; Mancuso, 2017). The potential toxic effect due to an over activation of the HO/BVR system has also been supported by the discovery of the importance of HO-1 gene repression in order to preserve cell homeostasis and integrity (Shibahara, 2003; Shibahara et al., 2003; Palozza et al., 2006). For this reason, the long-lasting induction of HO-1 due to chronic supplementation with herbal products may be a double-edged sword and the possibility of neurotoxicity must be carefully considered.

Regarding the effective dose correlation *in vitro* and blood or tissue concentrations found *in vivo*, resveratrol is an indisputable example. Both the bioavailability data through wine consumption and the EFSA opinion on anti-inflammatory efficacy do not justify the turmoil often generated by the media regarding the beneficial properties of resveratrol in red wine. In this regard, the antioxidant, antithrombotic, and metabolic effects of ethanol *per se* often are not mentioned; moreover, red wines usually contain ethanol in greater concentration than white wines and the beneficial effects claimed for the former can be due, once again, to ethanol (Goldberg et al., 1995; Criqui, 1998; Belleville, 2002). Focusing on the beneficial effects of moderate ethanol consumption would also allow a greater responsibility on the consumers’ part towards their own health, because of the pathological effects of uncontrolled alcohol intake are well known. On the contrary, if attention is focused on resveratrol, of which only the beneficial effects are artificially advertised, the need for a controlled intake of red wine is lost since the attention is driven towards a natural compound.

The risk linked to the effects on drug metabolizing enzymes and the health consequences in case of concomitant drug intake, concepts that are very often neglected also by health professionals, should be emphasized. Particularly dangerous are the interactions between *G. biloba* and CYP or other phase II enzymes, which could increase the risk of toxicity in patients treated with drugs, such as valproic acid, trazodone, talinolol, warfarin, etc. It is worth mentioning that the lack of reliable data on the neuroprotective effects of resveratrol and *G. biloba* challenges their potential beneficial effects in the treatment of neurodegenerative diseases. In this regard, more attention is required for FA for which, to our knowledge, there are no clinical studies confirming the promising neuroprotective results obtained on preclinical models. The few clinical studies available, addressing the kinetics in most cases, have been performed using foods rich in FA and this does not always allow to ascertain the actual dose administered and any confounding effects related to other active compounds present. Actually, only one randomized and double-blind study on hyperlipidemic patients (Bumrungpert et al., 2018) has appeared in the literature and shows the hypolipemic, anti-inflammatory, and antioxidant effects of FA (1 g/day *per os* for 6 weeks).

These considerations lead to the conclusion that there is not sufficient evidence on the efficacy of these herbal products in neurodegenerative diseases and that further efforts and many attempts are recommended and requested by doctors, researchers, and other health professionals to bridge this gap for the benefit of patients and their families.

## Author Contributions

Conceptualization: CM, VC, LT. Data curation: CM, EM. Writing–original draft preparation: CM, EM. Writing–review and editing: CM, EM, VC, LT.

## Conflict of Interest

The authors declare that the research was conducted in the absence of any commercial or financial relationships that could be construed as a potential conflict of interest.
